# Radiation Retinopathy: Case report and review

**DOI:** 10.1186/1471-2415-7-6

**Published:** 2007-04-05

**Authors:** Abha Gupta, Felipe Dhawahir-Scala, Amy Smith, Lorna Young, Steve Charles

**Affiliations:** 1 Manchester Royal Eye Hospital, Lister House, Oxford Road, Manchester, UK, M13 9WH

## Abstract

**Background:**

Ocular damage from radiation treatment is a well established phenomenon. Many factors are now known to influence the incidence of radiation retinopathy, including total dosage and daily fraction size. Patients who are diabetic, hypertensive or received previous chemotherapy are more susceptible to radiation retinopathy.

**Case Presentation:**

A 55 year old male was referred from the oncology department with epiphora. His medical history included Type 2 Insulin treated Diabetes Mellitus and hypertension. One year prior to presentation he had undergone a total rhinectomy with a 4 week course of post-operative radiotherapy for an aggressive sqaumous cell carcinoma of the nose. On examination the visual acuity was noted to be 6/36 left eye and 6/9 right eye. Posterior segment examination revealed marked retinal ischaemia present in the posterior pole and macular region of both eyes. The appearance was not thought to be typical of diabetic changes, radiation retinopathy being the more likely diagnosis especially in view of his history. Over the next four months the vision in both eyes rapidly deteriorated to 3/60 left eye and 1/60 right eye. Bilateral pan retinal photocoagulation was thought to be appropriate treatment at this point.

**Conclusion:**

This case highlights the importance for ophthalmologists and oncologists to be aware of the close relationship between diabetes and radiation treatment and the profound rapid impact this combination of factors may have on visual function. Radiation is being used with increasing frequency for ocular and orbital disease, because of this more cases of radiation retinopathy may become prevalent. Factors which may potentiate radiation retinopathy should be well known including, increased radiation dosage, increased fraction size, concomitant systemic vascular disease and use of chemotherapy. Counselling should be offered in all cases at risk of visual loss. As no effective treatment currently exists to restore visual function, monitoring of visual acuity in all cases and early referral to the ophthalmologist as appropriate is warranted.

## Background

Ocular damage from radiation treatment is a well established phenomenon. Many factors are now known to influence the incidence of radiation retinopathy, including total dosage and daily fraction size. Patients who are diabetic, hypertensive or who have received previous chemotherapy are more susceptible to radiation retinopathy. In this article we describe a case of a man who developed fulminant radiation retinopathy despite adequate precautions, due to the confounding problem of diabetes. We also discuss the current literature on radiation retinopathy.

## Case Presentation

**Figure 1:**
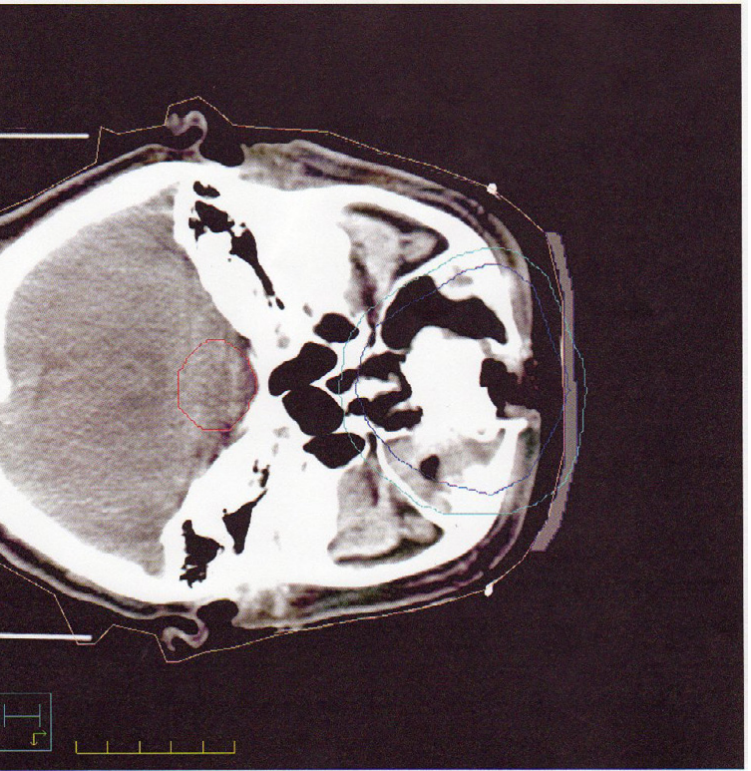
CT Scan showing area of sqaumous cell carcinoma.

A 55 year old male was referred from the oncology department with epiphora. His medical history included type 2, Insulin treated, Diabetes Mellitus of 10-years duration and controlled hypertension. He smoked between 20–40 cigarettes per day. One year prior to presentation he had undergone a total rhinectomy with a 4 week course of post-operative radiotherapy for an aggressive sqaumous cell carcinoma of the nose (see figure 1). He received a total of 5250 rad (52.5 gray) to the tumour (1312.5 rad/13.12 gray per week, 262.5 rad/2.62 gray per session). Since treatment there had been no evidence of tumour recurrence. He underwent diabetic eye screening through his optometrist and a diabetic retinal check in April 2005 was recorded as showing no diabetic eye disease. There was no other ocular history of note.

**Figure 2:**
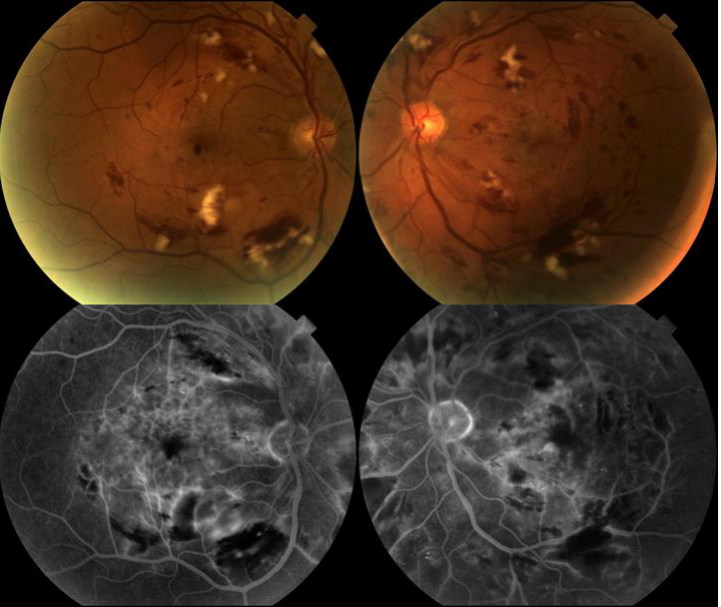
**Radiation Retinopathy at initial presentation**. The colour fundus photographs above show clinical evidence of marked bilateral ischaemic changes. The fluorescein photographs show evidence of masking from the haemorrhages and also areas of capillary dropout reflecting ischaemic areas.

**Figure 3:**
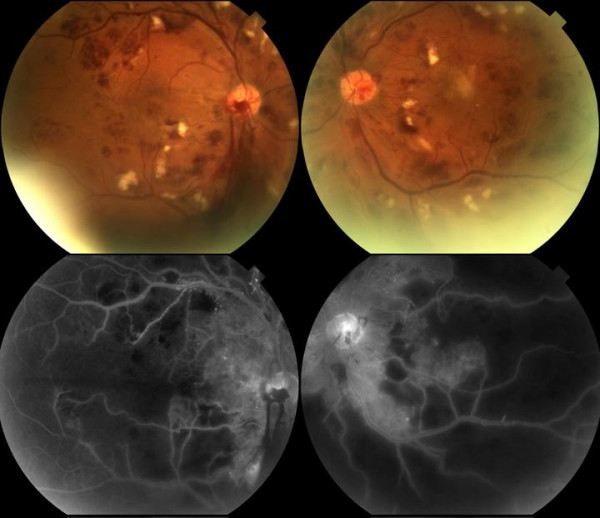
**Radiation retinopathy prior to laser treatment**. The photographs above show increasing severity of the radiation retinopathy with evidence of bilateral vitreous haemorrhage secondary to disc neovascularization. Severe ischaemic areas and areas of non-viable peripheral retina are visible, an area of viable ischemic retina along the arcades close to the disc is thought to be responsible for the disc noevascularisation.

On examination the visual acuity was noted to be 6/36 left eye and 6/9 right eye. He had bilateral medial lower lid ectropion accounting for the epiphora. Anterior segment examination and intraocular pressures were normal. Posterior segment examination revealed marked retinal ischaemia present in the posterior pole and macular region of both eyes. Fundus fluorescein angiography (FFA) confirmed an ischaemic retina. There were no signs of neovascularization (see figure 2). The distribution of the retinal changes with severe ischaemic changes in the posterior retina more than in the anterior retina, the relative lack of microaneurysms compared to the high numbers of cotton wool spots and blot haemorrhages and the rapid nature in which the retinopathy developed made the diagnosis of radiation retinopathy likely. Additionally the temporal onset of the retinopathy in relation to the radiotherapy (1 year post treatment) and the dose delivered further supported this diagnosis. Over the next four months the vision in both eyes rapidly deteriorated to 3/60 left eye and 1/60 right eye. Clinical examination revealed bilateral vitreous haemorrhage. FFA confirmed bilateral disc neovascularisation and evidence of a grossly ischaemic retina with little residual viable retina (see figure 3). Bilateral pan retinal photocoagulation was considered to be appropriate treatment at this stage as it was thought that the minimal remaining, highly ischaemic, retina close to the arcades may be responsible for the disc neovascularisation. The total area of treatment in RE was 2595 mm^2 ^and in the LE 1825 mm^2^. At review in September 2006 the visual acuities were counting fingers in the RE and 6/60 aided in the LE. The retinopathy is stable.

## Conclusion

The clinical features of radiation retinopathy include microaneurysms, cotton wool spots, capillary dilation, telangectasia, and capillary closure. The posterior retina is more sensitive to radiation than the peripheral retina [1-3]. Vascular compromise may result in retinal oedema. Ischaemia may lead to disc neovascularization which in turn can cause vitreous haemorrhage and retinal detachment. Histologically there is thickening of arteriolar and capillary walls and loss of endothelial cells [1-3]. Histologically these findings differ from diabetic retinopathy in that there is early loss of endothelial cells in radiation retinopathy compared to diabetic retinopathy where pericytes are affected initially. There are also less number of microaneurysms present compared to diabetic retinopathy, this was noted in our patient favouring a diagnosis of radiation retinopathy [1,3].

The total dose of radiation, along with the fraction size are important in the development of retinopathy[1-4]. A reported safe dose is 3000 rads/30 Gray, 1000 rads/10 Gray per week in five fractions (200 rads/2 Gray per session) [3], although cases have been reported with lower doses of radiotherapy [2,4]. One study reported 50% of patients developed retinopathy following 60 Gray [5] of radiation whilst another report showed a rise in incidence to 85–95% in those exposed to 70–80 Gray [6]. The time of onset of radiation retinopathy is between 6 months – 3 years, again it has been known to occur earlier or later [2]. This case received a higher than recommended radiation dose due to the aggressive nature of the carcinoma. It is likely that this combined with pre-existing diabetes resulted in the severe and rapid progression of retinopathy one year after treatment.

Factors which are known to exacerbate radiation retinopathy include chemotherapy and vascular diseases such as diabetes and hypertension[3,7-9]. Pregnancy has been thought to accelerate radiation retinopathy [10] and is also known to aggravate diabetic retinopathy [11].

Spontaneous improvement can occur but this is infrequent [12]. Mostly treatment with pan retinal photocoagulation is implemented when neovascularization is visible [1]. One study found 91% of patients treated with PRP for proliferative radiation retinopathy had regression of new vessels [13].

Ocular manifestations of radiotherapy are well known and include dry eye, epiphora, ectropion, scleral necrosis, cataract, glaucoma, optic neuropathy and retinopathy [14] Cataracts and radiation retinopathy are the most common visually limiting complications seen after ophthalmic plaque radiation therapy [15]. The cataracts are amenable to surgical treatment mostly leading to improvement in vision. However, retinopathy can lead to permanent and severe visual loss. Currently no guidelines or treatment exists for radiation retinopathy. Pan Retinal Photocoagulation is performed in the proliferative stage in an attempt to prevent further visual loss, although studies have shown that earlier intervention may be more beneficial in preserving vision [16]. There are studies which have reported a temporary improvement in vision after using intravitreal triamcinolone [17,18] or focal laser treatment for cases with radiation maculopathy [19].

Recently a classification has been devised by Finger and Kurli [14] which describes stages of radiation retinopathy in relation to the clinical signs seen, symptoms, location, best method of visualization and the risk of vision loss. This is important as there is a need for common language for this retinopathy for future comparative studies. Our patient was initially asymptomatic and found to have cotton wool spots, retinal haemorrhages, exudates, choriodopathy and retinal ischaemia involving posterior pole and peripheral retina. These findings relate to stage 2 of Finger's radiation retinopathy classification, stage 2 carries a guarded prognosis for vision having a moderate risk of visual loss. The patient rapidly progressed over 4 months to stage 4, where visual loss did occur and retinal neovascularisation along with vitreous haemorrhage was present.

Finger et al suggest that early pan retinal photocoagulation is useful in inducing regression of radiation retinopathy and also that treatment before clinically apparent radiation retinopathy is present may be more effective than treatment after its onset, especially in high risk cases. This is especially important in cases of radiation maculopathy as prevention is more likely to preserve vision than treatment after its onset. This results of this study also suggest that treatment of cases with no clinical evidence of retinopathy were less likely to develop maculopathy post laser treatment. This highlights the need for monitoring visual acuity post radiation treatment, especially in high risk cases [16].

This case highlights the importance for ophthalmologists and oncologists to be aware of the close relationship between diabetes and radiation treatment and the profound rapid impact this combination of factors may have on visual function. It is unlikely that the rapid progression of retinopathy and dramatic loss of vision in this patient was due to diabetes alone. He was not noted by his optician to have any prior diabetic retinopathy and at his blood pressure was well controlled medically. Diabetes and radiation primarily damage the retinal capillaries and thus a potentiating effect is not surprising. Diabetes results in early loss of pericytes and thickening of the basement membrane. Radiation however, damages the endothelial cells. As endothelial cells and pericytes are the primary cells making retinal capillaries damage to these cells, through a combination of diabetes and radiation, leaves little cellular support. This would result in the visible changes of capillary closure, vessel leakage, aneurysms and haemorrhage.

Radiation is being used with increasing frequency for ocular and orbital disease, because of this more cases of radiation retinopathy may become prevalent. Factors which may potentiate radiation retinopathy should be well known including, increased radiation dosage, increased fraction size, concomitant systemic vascular disease and use of chemotherapy (See Table 1).

**Table 1:** Factors potentiating radiation retinopathy

Increased radiation dosage
Increased Fraction size
Systemic vascular disease
Chemotherapy

Counselling should be offered in all cases at risk of visual loss. In some cases it may not be possible to protect the eyes during radiation, in these cases one should be aware of the factors which may potentiate eye disease, the dose and area of the retina irradiated should be minimized.

As no effective treatment currently exists to restore visual function, monitoring of visual acuity in all cases and early referral to the ophthalmologist as appropiate is warranted.

Further studies should be performed in order to produce treatment guidelines for radiation retinopathy. Clinical trials also need to be performed to establish whether early PRP is beneficial in reducing the onset of radiation retinopathy in eyes at risk and also to determine if early PRP is useful in inducing regression of established radiation retinopathy and improve visual outcomes.

## Competing interests

The author(s) declare that they have no competing interests.

## Authors' contributions

AG drafted the manuscript, did a literature review, discussed the case with the oncologists and wrote consent form

FDS examined and managed the patient, consented patient for the article to be published, did the initial literature review and critically analysed manuscript

AS examined the patient and analysed revisions of the manuscript

LY offered valuable insight into the diagnosis and management of this case

SC offered valuable insight into the diagnosis and treatment of this case

All authors have read and approved the final manuscript

## Pre-publication history

The pre-publication history for this paper can be accessed here:


